# Hebbian Plasticity in CPG Controllers Facilitates Self-Synchronization for Human-Robot Handshaking

**DOI:** 10.3389/fnbot.2018.00029

**Published:** 2018-06-08

**Authors:** Melanie Jouaiti, Lancelot Caron, Patrick Hénaff

**Affiliations:** ^1^Université de Lorraine, CNRS, Inria LORIA, Nancy, France; ^2^Information and Systems Department, Ecole Nationale Supérieure des Mines de Nancy, Nancy, France

**Keywords:** physical human robot interaction, hebbian learning, central pattern generator (CPG), adaptive behavior, handshaking, plasticity, neural oscillators

## Abstract

It is well-known that human social interactions generate synchrony phenomena which are often unconscious. If the interaction between individuals is based on rhythmic movements, synchronized and coordinated movements will emerge from the social synchrony. This paper proposes a plausible model of plastic neural controllers that allows the emergence of synchronized movements in physical and rhythmical interactions. The controller is designed with central pattern generators (CPG) based on rhythmic Rowat-Selverston neurons endowed with neuronal and synaptic Hebbian plasticity. To demonstrate the interest of the proposed model, the case of handshaking is considered because it is a very common, both physically and socially, but also, a very complex act in the point of view of robotics, neuroscience and psychology. Plastic CPGs controllers are implemented in the joints of a simulated robotic arm that has to learn the frequency and amplitude of an external force applied to its effector, thus reproducing the act of handshaking with a human. Results show that the neural and synaptic Hebbian plasticity are working together leading to a natural and autonomous synchronization between the arm and the external force even if the frequency is changing during the movement. Moreover, a power consumption analysis shows that, by offering emergence of synchronized and coordinated movements, the plasticity mechanisms lead to a significant decrease in the energy spend by the robot actuators thus generating a more adaptive and natural human/robot handshake.

## 1. Introduction

For humans, physical and social interpersonal interactions induce gestural and verbal/non-verbal communications based on rhythmic mechanisms and rhythmic movements. These mechanisms and the associated synchronization phenomena (limit cycles and clamping) could play a fundamental role in physical and social interpersonal interactions (Troje et al., 2006; Yonekura et al., 2012) and could be an emergent feature of the physical and social interactions between humans who adapt to each other and learn from each interaction, generating synchronization phenomena and creating conscious or unconscious links between people (Delaherche et al., 2012). Scientists assume that emotional and social interactions involve a coupling between individuals which is achieved thanks to neural structures with similar properties as those implicated in the neural control of movements. For example, coordination of oscillatory motions between two individuals (two distinct brains) obeys the same rules as for inter-limb coordination within a single individual (single brain) (Schmidt et al., 1990; Tognoli et al., 2007). Thus, distinct individuals can spontaneously interact and successfully perform coordinated actions through an exchange of information by means of their sensorimotor, cognitive and social underpinnings.

In humans and animals, rhythmic movements rely on universal sensory-motor mechanisms (Cruse et al., 1998; Cattaert and Le Ray, 2001; Zehr et al., 2004) and result from learning processes implying chaotic neural oscillators in central pattern generators (CPGs). CPGs endowed with plasticity rules allowing for synchronization with the control body (Shadmehr, 2010), are also implied both in the generation of discrete and rhythmic movements (Grillner, 2006).

In human interactions, handshaking is an important and universally social function allowing social introduction in various contexts, regulating and maintaining human interactions (Schiffrin, 1974; Hall and Spencer Hall, 1983; Bernieri and Petty, 2011; Giannopoulos et al., 2011) but it can also provide information on the health and emotional state of a person (Chaplin et al., 2000), which could be useful for assistive robotics. It is a multimodal physical interaction, socially common but complex to reproduce with a humanoid robot because it involves fine and complex movement coordination which engages the body and gaze throughout the act: from the preparation to the contact, the locking, the rhythmic and synchronized movement until the withdrawal of the hands (Walker et al., 2013). How synchronized motion of two humans arms is established and maintained is still an open question but some aspects have been studied in the movement science and neuroscience fields, such as reaching hands (Lee, 1976; Bastin et al., 2006) and interpersonal synchronization tasks (Oullier et al., 2008; Dumas et al., 2014).

From a neuroscience point of view, handshaking implies interpersonal motor coordination and recent research showed that it also induces the synchronization of the brain activity of both partners Tognoli et al. (2007). Therefore, it can be considered as a paradigm for social and physical interactions, in particular because its multimodality is based on physical and social clamping of rhythmic movements. Consequently, if we want humanoid robots to be able to interact properly with humans, i.e., in a socially acceptable way, shaking hand with humans like a human is an interesting challenge (Der and Martius, 2017). It is then necessary to design bio-inspired robot controllers able to produce rhythmic movements and trigger the emergence of a synchronization in an interaction such as the handshaking gesture. One possible way to achieve this consists in designing robot controllers which are intrinsically rhythmic, such as CPGs, but which also incorporate synchronization learning abilities similarly to the plasticity mechanisms involved in the human motor nervous system for rhythmic movement production.

Several models of CPGs have been proposed for many years in order to understand human and animal motor control mostly aiming at locomotion control in robotics (Ijspeert, 2008; Yu et al., 2014; Nachstedt et al., 2017). CPGs are neuronal structures located in the spinal cord and able to generate rhythmic and discrete activities that can be initiated, modulated and reset by different kinds of signals: descendant signals from high level structures located in the MLR (mesencephalic locomotor region) (Grillner, 2006; Rossignol et al., 2006; Harris-Warrick, 2011) or afferent sensory feedbacks coming from low levels of the body (proprioceptive) or from the environment (exteroceptive) (Marder and Calabrese, 1996; Pearson, 2004). Different levels of CPG modeling exist from the microscopic level (called also biophysical model) that takes into account many details in the biophysical operation of the neurons like the famous Hodgkin-Huxley model (Hodgkin and Huxley, 1952), to the macroscopic level that tries to reproduce the functionality of a population of neurons using non-linear oscillators like Van der Pol (Rowat and Selverston, 1993; Low et al., 2006), Rayleigh (Mottet and Bootsma, 1999), or Hopf (Righetti and Ijspeert, 2006; Nachstedt et al., 2017).

Between the microscopic and macroscopic levels of modeling, there exists an intermediary level, called mesoscopic level, which takes a more realistic biological inspiration but is sufficiently simplified to study the sensorimotor couplings, oscillation properties and learning mechanisms involved in the control of rhythmic tasks. These models are usually based on a pair of two mutually inhibitory oscillating neurons thus creating a CPG, called half-center (Grillner and Wallen, 1985), divided into two parts controlling the extensor and flexor muscles.

The model of half-center CPG for mammal locomotion by McCrea and Rybak (Rybak et al., 2006) takes inspiration from biological structures, such as the rhythmic layer, modulating layer, interneurons, sensory neurons, etc. Its architecture is divided into three layers: Rhythm Generator layer (composed of an inhibitory pair of oscillatory neurons), Pattern Formation layer (composed of inter-neurons) and Motor layer (composed of Motoneurons). It also takes afferent (proprioceptive) and efferent (exteroceptive) sensory feedbacks into account. While this model has been widely used for locomotion (Amrollah and Henaff, 2010; Spardy et al., 2011; Nassour et al., 2014; Danner et al., 2016; Nachstedt et al., 2017), very few works apply it to the control of upper limb movements: to our knowledge, only Teka et al. (2017) used it to study the reaching movement.

Non-linear oscillator models (also called relaxation-oscillators) can be used for oscillating neurons in CPGs because they can synchronize effortlessly with an external signal provided the frequency of this signal is not too different from the intrinsic frequency of the oscillator (Pikovsky et al., 2003; Petrič et al., 2011). Thus, non-linear oscillators are suitable models to explain and reproduce the synchrony phenomena that emerge in interpersonal coordination, especially if they are implemented at the rhythmic level of a CPG. In this case, by acting like a dynamic attractor, they facilitate the self-synchronization of the CPG with the dynamic of the limb controlled by the CPG.

During the production of movement coordination, the Matsuoka oscillating neuron model (Matsuoka, 1987) exhibits the behavior of a non-linear oscillator and self-synchronization. This model has been used extensively in robotic locomotion or human motor control modeling (Taga et al., 1991; Taga, 1998; Kasuga and Hashimoto, 2005; Degallier and Ijspeert, 2010; Yu et al., 2014; Avrin et al., 2017a,b). However, the main problem of the Matsuoka model is that it cannot produce discrete as well as rhythmic activities as mentioned in Degallier and Ijspeert (2010). Indeed, it is now known that, in motor control, discrete and rhythmic movements are generated by networks of spinal neurons (Grillner, 2006; Degallier et al., 2011). Consequently, in order to be biologically plausible, a CPG model must be able to produce both discrete and rhythmic activities, just like what has been observed in biological neurons implied in locomotion production (Marder and Bucher, 2001). Therefore, CPGs must include oscillating neurons able to operate in discrete and rhythmic modes depending on one or several parameters. Unfortunately, although the Matsuoka model is a non linear oscillator, its nonlinearity is not controllable, meaning the model doesn't have a nonlinear parameterizable function allowing different nonlinear behaviors.

The Rowat-Selverston oscillating neuron model (Rowat and Selverston, 1993) is able to produce discrete and rhythmic activities depending of two parameters as it has been demonstrated in Amrollah and Henaff (2010) and Nassour et al. (2014). However, only a few studies make use of it (Arikan and Irfanoglu, 2011). The Rowat-Selverston oscillating neuron is a generalized Van der Pol oscillator and consequently all known properties of the Van der Pol can be applied to it, especially the dynamic Hebbian learning of frequency introduced by Righetti et al. (2006).

The first originality of this article is to implement Hebbian mechanisms proposed by Righetti et al. (2006), in a bio-inspired CPG, which we previously used for biped locomotion (Nassour et al., 2014), enabling it to learn to synchronize with an external signal. The second originality resides in using this plastic CPG to control a simulated robotic arm which has to learn to synchronize its oscillatory movements with the frequency of an external force applied to its effector, thus reproducing the act of handshaking with a human.

In the first part, we explain how dynamic plasticity is integrated in our CPGs and present the design of our robot controller. In the second part, we validate our model by applying it to the command of a robotic arm interacting physically rhythmically in simulation. We show that the controller learns to synchronize with the imposed rhythm in a given frequency range matching the usual frequencies of handshaking. We also demonstrate the importance of plasticity to achieve fast and stable coordination. In the fourth part, we discuss our results and future prospects.

## 2. Materials and methods

This section presents the plasticity mechanisms implemented in the neurons of the CPG and finally, the design of the CPG-based controller.

### 2.1. Dynamic plasticity in CPGs based on rowat-selverston neurons

As mentioned above, a non-linear oscillator has the property of self-synchronization with an oscillating external signal applied as its input, provided the frequency of this signal is close enough to the intrinsic frequency of the oscillator. Implementing frequency learning mechanisms inside a CPG would allow to synchronize its rhythmic activity with the external signal even if the frequency of this signal is significantly different from the intrinsic one of the CPG (Ijspeert, 2008; Yazdani et al., 2017). Therefore, the CPG could synchronize with the movements, triggering the emergence of a global coordination between the limbs (Degallier and Ijspeert, 2010). Righetti et al. (2006) proposed such a frequency learning model for a Van der Pol oscillator called Dynamic Hebbian learning. This section demonstrates the application of this idea to the Rowat-Selverston oscillating neuron model.

#### 2.1.1. Recall of righetti's model for dynamic hebbian learning into van der pol oscillators

The free form (i.e., without any input signal applied) of the Van der Pol oscillator can be written as :

(1) $$\begin{array}{l} \dot{x}=y\\ \dot{y}=-\alpha \left(x^{2}-p\right)y-\omega^{2}x \end{array}$$

where *y* is the output of the oscillator, *p* amplitude of *y*, α controls the degree of nonlinearity of the system and ω mainly influences the frequency of the oscillator.

When the Van der Pol oscillator is forced by an oscillating input signal *F*(*t*) the model can be written as:

(2) $$\begin{array}{l} \dot{x}=y+\epsilon F\\ \dot{y}=-\alpha \left(x^{2}-p\right)y-\omega^{2}x \end{array}$$

where ϵ can be seen as a gain or a weight.

In order to synchronize the oscillator with the input *F*(*t*) (see Righetti et al., 2006 for details), proposed to learn the frequency of the oscillator following a Hebbian learning rule :

(3) $$\begin{array}{r} \begin{array}{cc} \dot{\omega } & =\epsilon F\frac{y}{\sqrt{x^{2}+y^{2}}} \end{array} \end{array}$$

They showed that this rule allows the oscillator to change its intrinsic frequency to synchronize with the oscillating signal *F*(*t*). The oscillator preserves the learned frequency, even after the input signal is cut. It has been applied to the Hopf oscillator and the Fitzhugh-Nagumo oscillator.

#### 2.1.2. Van der pol form of rowat-selverston neuron

The free form of the Rowat-Selverston model of a cellular neuron is described by the equations (see Rowat and Selverston, 1993 for details):

(4a) $$\begin{array}{r} \tau_{m}\dot{V}+V-A_{f}\tanh\left(\frac{\sigma_{f}}{A_{f}}V\right)+q=0 \end{array}$$

(4b) $$\begin{array}{r} \tau_{s}\dot{q}=-q+\sigma_{s}V \end{array}$$

with *V* being the cellular membrane potential, *q* the slow current, τ_*m*_ the time constant of the cellular membrane, τ_*s*_ is the time constant of slow current activation (τ_*m*_≪τ_*s*_), σ_*s*_ and σ_*f*_ represent respectively the conductance of slow and fast currents, *A*_*f*_ influences the amplitude of *V*.

Because Rowat-Selverston is a generalized Van der Pol oscillator, its equations can be rewritten in a Van der Pol form such as in Equation (1). To do that, Equation (4a) can be differentiated, and $\dot{q}$ replaced by the expression given in Equation (4b).

We can thus obtain a new expression of the unforced Rowat-Selverston oscillator. In order to identify a Righetti learning rule in the Rowat-Selverston neuron model, we must liken this model to a Van der Pol oscillator expressed by Equation (1). To do that, we approximate the tanh function to a linear one, tanh(*x*)≈*x*, thus yielding:

(5) $$\begin{array}{r} \tau_{m}\ddot{V}+\left(\frac{\tau_{m}}{\tau_{s}}+1-\sigma_{f}+\frac{\sigma_{f}^{3}}{A_{f}^{2}}V^{2}\right)\dot{V}+\frac{1+\sigma_{s}}{\tau_{s}}V-\frac{\sigma_{f}}{\tau_{s}}V=0 \end{array}$$

We're well aware that approximating tanh(*x*) to *x* may seem far-fetch and exceedingly inaccurate. Here, we are only trying to identify a Hebbian rule and experiments validate our attempt. It may very well be that, other rules, based on other far-fetched assumptions, are valid too.

By setting, $\dot{V}=y$, we can transform the model into the following unforced Van der Pol form see the Appendix for the detailed calculations:

(6) $$\begin{array}{l} \dot{V}=y\\ \dot{y}=\frac{-\sigma_{f}^{3}}{\tau_{m}A_{f}^{2}}\left(V^{2}-\frac{A_{f}^{2}(\sigma_{f}\tau_{s}-\tau_{m}-\tau_{s})}{\tau_{s}\sigma_{f}^{3}}\right)y-\frac{1+\sigma_{s}-\sigma_{f}}{\tau_{s}\tau_{m}}V \end{array}$$

By comparing this equation to Equation (1), we can finally identify the Van der Pol parameters ω, α and *p* of the unforced Rowat-Selverston oscillating neuron:

$$\begin{array}{rcl} \omega & = & \sqrt{\frac{1+\sigma_{s}-\sigma_{f}}{\tau_{s}\tau_{m}}};\alpha =\frac{\sigma_{f}^{3}}{\tau_{m}A_{f}^{2}}; \end{array}$$

(7) $$\begin{array}{rcl} p & = & \frac{A_{f}^{2}\left(\tau_{s}\left(\sigma_{f}-1\right)-\tau_{m}\right)}{\tau_{s}\sigma_{f}^{3}};\text{with}\sigma_{f}<1+\sigma_{s} \end{array}$$

#### 2.1.3. Implementing dynamic hebbian learning into the rowat-selverston neuron

When an external signal *F*(*t*) is applied to the Rowat-Selverston oscillating neuron, the neuron potential *V* becomes:

(8) $$\begin{array}{r} \dot{V}=y+\epsilon F \end{array}$$

where the gain ϵ can be considered like a synaptic weight. Thus, the principle of Hebbian dynamic rule proposed by Righetti et al. (2006) can be applied on the parameters of the Rowat-Selverston model to learn the frequency of *F*(*t*).

As shown in Rowat and Selverston (1993), the frequency of the neuron oscillations depends only on τ_*m*_, τ_*s*_, σ_*f*_, and σ_*s*_: if σ_*f*_ is fixed above a given threshold $\theta_{f}=1+\frac{\tau_{m}}{\tau_{s}}\approx 1\;\left(\tau_{m}\ll \tau_{s}\right)$, σ_*s*_ controls two modes depending on another threshold θ_*s*_. If σ_*s*_ < θ_*s*_, there are no oscillations [intrinsic mode called “plateau potentials” in Marder and Bucher (2001)]. On the other hand, for σ_*s*_>θ_*s*_, the neuron produces a rhythmic signal [intrinsic mode called “endogenous bursting” in Marder and Bucher (2001)] whose frequency depends on τ_*m*_, τ_*s*_, and σ_*s*_.

Following the idea of Righetti et al. (2006), we propose to implement dynamic Hebbian learning of the oscillations frequency by learning σ_*s*_ depending on the signal *F*(*t*) applied to the neuron and weighted by ϵ. Thus, neural plasticity for frequency learning can be obtained by deriving the expression of ω^2^ from 7 :

(9) $$\begin{array}{r} {\dot{\sigma }}_{s}=2\dot{\omega }\omega \tau_{m}\tau_{s}=2\dot{\omega }\sqrt{\tau_{m}\tau_{s}}\sqrt{1+\sigma_{s}-\sigma_{f}} \end{array}$$

By applying the dynamic Hebbian learning rule proposed by Righetti et al. (2006) to Equation (3), we obtain :

(10) $$\begin{array}{r} \dot{\sigma_{s}}=2\epsilon F\sqrt{\tau_{m}\tau_{s}}\sqrt{1+\sigma_{s}-\sigma_{f}}\frac{y}{\sqrt{V^{2}+y^{2}}};\sigma_{f}<1+\sigma_{s} \end{array}$$

We can see that this learning rule depends on the CPG time constants. The presence of σ_*s*_ on the right side of the equation, makes it a closed loop ensuring that the end value of σ_*s*_ does not depend on its initial value.

#### 2.1.4. Plasticity for afferent and efferent signals

Additionally, to improve the control realized by the CPG, we propose to learn the amplitude of neuronal oscillations by learning *A*_*f*_ depending on *F*(*t*), and to maintain the strength of sensitivity of *F*(*t*) efficient enough with a learning mechanism of ϵ.

##### 2.1.4.1. Neuronal plasticity for amplitude learning

*A*_*f*_ determines the amplitude of the output of the CPG (efferent signal) and thus the amplitude of velocity orders applied to the motors. When *A*_*f*_ is high, σ_*s*_ will oscillate globally before reaching stability. In Equation 4, the expression $A_{f}\text{tanh}\left(\frac{\sigma_{f}}{A_{f}}V\right)$ influences the amplitude of *V* and consequently the CPG output. If the amplitude is too big, the CPG becomes unstable due to the rapid switchings of the sigmoid function of interneurons located in the pattern formation layer, and if it is too small, the output of the CPG doesn't have enough energy. Adapting the amplitude of the neuron oscillations in accordance with the applied signal *F*(*t*) could solve that. One solution consists in minimizing the error between the quadratic values of *F*(*t*) and the argument of tanh() in equation 4 to match the amplitude of *V* with *F*(*t*) :

(11) $$\begin{array}{r} \dot{A_{f}}=-\mu \left({\left(\nu \frac{\sigma_{f}V}{A_{f}}\right)}^{2}-F^{2}\right) \end{array}$$

where ν is a scale factor and μ a learning step.

The presence of *A*_*f*_ in the equation makes it a closed loop, guarantying the same end value for *A*_*f*_ no matter the initial value. Empirically, we found that 20 was the best value for ν. Since *A*_*f*_ is not a constant any more, its derivative should appear in equation 5. This case was studied and the same result with an additional term $\frac{V^{3}\sigma_{f}^{3}}{A_{f}^{3}}{Ȧ}_{f}$ was obtained. Ȧ_*f*_ being extremely small, this last term can be neglected and thus, yields the same result. So this case won't be detailed here any further.

##### 2.1.4.2. Synaptic plasticity for sensitivity learning

ϵ acts like a learning step for σ_*s*_ (local oscillation) and determines how much σ_*s*_ will oscillate when a new input signal is applied before reaching stability. As a consequence, a small ϵ will allow for a more robust and stable learning but will require more time to reach stability, if the interaction doesn't last long enough, it may never be reached. On the other hand, a large ϵ will lead to a more unstable learning but the final σ_*s*_ may be reached. The parameter ϵ also acts as a synaptic weight to the afferent signal *F*(*t*) that feedbacks to the CPG. In Equation 11, we're really only interested in the frequency of *F*(*t*) and its magnitude is not relevant.

So, ϵ can be considered as a synaptic weight that could enable the CPG to better sense the external signal *F*(*t*) by normalizing it to magnitude 1. Besides, it was empirically determined that if ϵ*F* is too small (< 1), σ_*s*_ changes are too slow and may never reach a stable final value and when ϵ*F* is too big (>1), σ_*s*_ becomes unstable. Optimal results are obtained when the amplitude of product ϵ*F* equals 1. From there, a learning equation of ϵ can be based also on an error of quadratic values pondered by a variable gain that limits extreme values of *F*(*t*):

(12) $$\begin{array}{r} \dot{\epsilon }=\lambda \tanh\left(\xi F\right)\left(1-{\left(\epsilon F\right)}^{2}\right) \end{array}$$

with ξ an empirically determined gain ensuring that the term inside the tanh is big enough (in our case, ξ = 100 yields good results). This term guaranties that learning occurs only when *F*(*t*) is not zero.

Here, it could be argued that there is no need for learning ϵ, that manually determining the optimal value of ϵ beforehand would be sufficient. By all means, this could be done but the system would be less versatile and this would be ignoring the fact that the amplitude of the input varies over time. Even if the amplitude seldom varies so drastically, so that the ϵ wouldn't be valid any more, it isn't the optimal value for ϵ and the system could be performing better, especially if the input signal varies over time. In that case, ϵ would be suitable for a range of frequencies but if the frequency becomes too low or too high, the system won't behave as expected, thus requiring an adaptive ϵ.

### 2.2. Designing the CPGs-based controller

**Figure 1:**
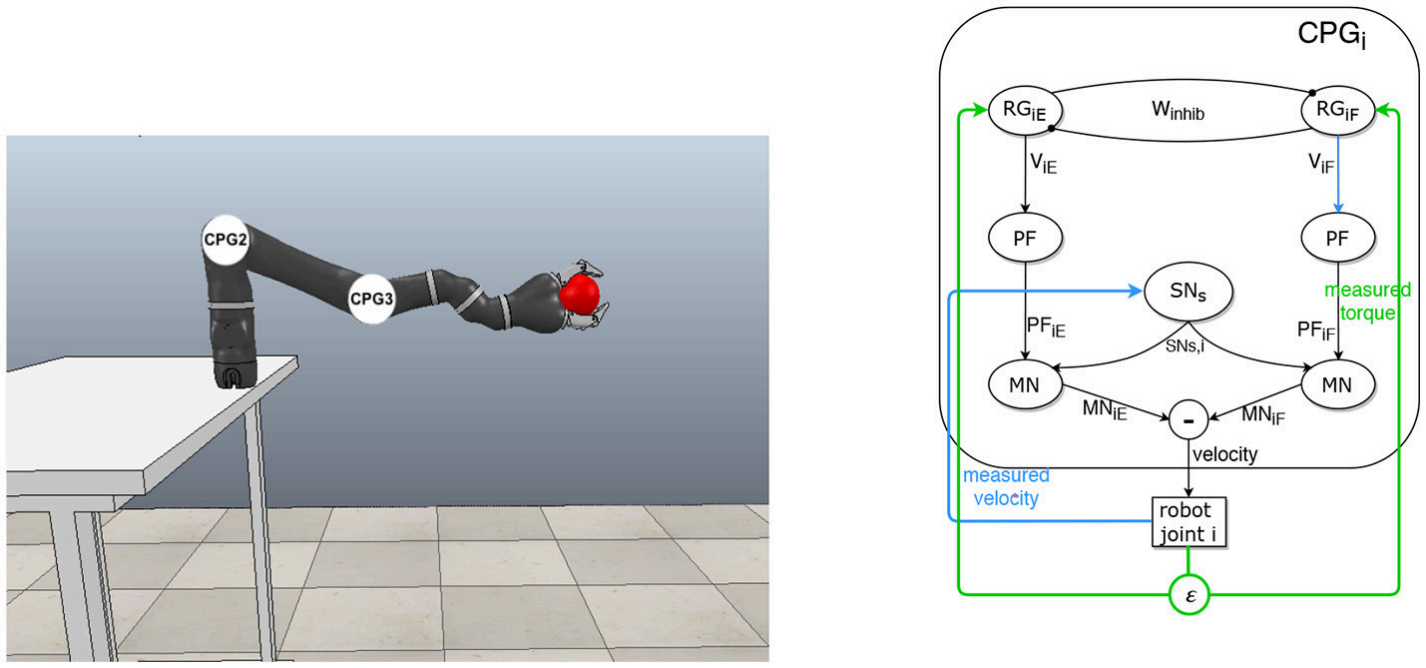
Principle and details of the CPG architecture for controlling the robotic arm. **(Left)** Simulation of the robot arm that interacts physically with a “virtual human hand” simulated by a ball in motion. Each CPG controls one joint motor velocity. **(Right)** Generic CPG for one joint and its afferent feedbacks from the robot.

An architecture based on CPGs is designed, according to the McCrea and Rybak model, to control a robot interacting physically with a human partner. The robot is a Mico robotic arm from Kinova company (Figure 1). One CPG controls the joint motor by applying velocity orders (efferent signals) and receives proprioceptive feedbacks (afferent signals) from the joint: torque and velocity (Figure 1). The equations for the generic CPG are the following, with *i*∈ℕ, designating the joint id.

For the coupled Rhythm Generator cells:

(13) $$\begin{array}{rcl} {\dot{V}}_{i_{\left\{E,F\right\}}} & = & y_{i_{\left\{E,F\right\}}}-W_{inhib}\frac{y_{i_{\left\{E,F\right\}}}}{1+e^{-4y_{i_{\left\{F,E\right\}}}}}+\epsilon_{i_{\left\{E,F\right\}}}F_{i} \end{array}$$

(14) $$\begin{array}{l} {\dot{y}}_{i_{\left\{E,F\right\}}}=\frac{1}{\tau_{m}}\left(\sigma_{f}-\frac{\tau_{m}}{\tau_{s}}-1-\sigma_{f}\tanh^{2}\left(\frac{\sigma_{f}}{A_{f_{i}}}V_{i_{\left\{E,F\right\}}}\right)\right)y_{i_{\left\{E,F\right\}}}\\ \;-\frac{1+\sigma_{s_{i_{\left\{E,F\right\}}}}}{\tau_{s}\tau_{m}}V_{i_{\left\{E,F\right\}}}+\\ \;\frac{A_{f_{i_{\left\{E,F\right\}}}}}{\tau_{s}\tau_{m}}\tanh\left(\frac{\sigma_{f}}{A_{f_{i_{\left\{E,F\right\}}}}}V_{i_{\left\{E,F\right\}}}\right) \end{array}$$

The term in *W*_*inhib*_ models the mutual inhibition between the rhythmic cells for the extensor and the flexor.

The terms σ_*s*__*i*__{*E, F*}___, *A*_*f*__*i*__{*E, F*}___, and ϵ_*i*_{*E, F*}__ are defined by Equations (11–13) respectively.

Inter-neurons of pattern formation layer (neuron PF), sensory neurons (neuron SN) for afferent feedbacks and motoneurons (neurons MN) for efferent signals, are defined as a sigmoid function (Debnath et al., 2014; Nassour et al., 2014):

(15) $$\begin{array}{rcl} PF\left(V_{i_{\left\{E,F\right\}}}\right) & = & PF_{i_{\left\{E,F\right\}}}=\frac{1}{1+e^{\frac{-V_{i_{\left\{E,F\right\}}}}{2}}} \end{array}$$

(16) $$\begin{array}{rcl} SN_{s}\left(v_{mes_{i}}\right) & = & SN_{i,s}=\frac{1}{1+e^{\alpha_{s}v_{mes}}} \end{array}$$

(17) $$\begin{array}{rcl} MN\left(PF_{i_{\left\{E,F\right\}}},SN_{i,s}\right) & = & MN_{i_{\left\{E,F\right\}}}=\frac{1}{1+e^{\alpha_{m}\left(PF_{i_{\left\{E,F\right\}}}-SN_{i,s}\right)}} \end{array}$$

With α_*s*_ = −0.061342 and α_*m*_ = 3. These coefficients were chosen to match the parameters of the robot. For instance, the sigmoid slope of the sensory neuron is determined by the range of values of the speed.

## 3. Simulation of human-robot handshaking: results

In this section, we will first present our results with a handshake simulation, then we will study the parameters influence and finally, we will demonstrate the importance of neuronal and synaptic plasticity.

The simulations have been run in the V-REP Simulation software with the Kinova Mico robotic arm. The V-REP simulator cannot realistically compute grasping with a human hand, so we simulate the handshaking gesture with a ball placed inside the gripper. The ball is defined as a static object not subjected to gravity that, unless stated otherwise, moves up and down according to a 2 Hz sinusoidal signal of amplitude 0.16 m. This frequency is coherent for handshaking according to previous experiments dedicated to the study on handshaking between humans (Tagne et al., 2016). Since both objects are collidable, the ball exerts a force on the fingers of the gripper, forcing the arm to move along (see Figure 1). Reaching and grasping details are irrelevant to this work and won't be detailed here.

The Mico arm has seven degrees of freedom, but Tagne et al. (2016) showed that arms are moving in the sagittal plan. In the current setup, only the shoulder and elbow (joints 2 and 3 of the Mico robot) are controlled for handshaking simulation, the five other joints are hence locked and unable to move. At the beginning of the simulation, the robot isn't subjected to any external force (other than gravity). The robotic arm raises toward the ball and grasps it. Then, by applying a sinusoidal signal to the ball, it must move in the vertical plane, thus applying a perturbation to the robotic arm. Finally, the ball is released and the interaction stops.

In all simulations here, the robotic arm raises toward the ball between *t* = 0 and *t* = 0.68*s*, then the interaction starts. The length of the interaction varies depending on the test conducted. Finally, when the ball is released, the behavior of the robotic arm is observed during ten more seconds before the simulation stops. Sensory feedbacks are taken into account during the whole process and are fed as an input to the CPG.

### 3.1. Role of feedbacks and mutual inhibition on plasticity

The choice of the parameters is a crucial step, when inappropriately chosen, the system may not behave as expected or the results may be subpar. So, in order to select the best parameters for the CPG, the role and influence of each parameter were studied.

To have an oscillating system, Rowat and Selverston (1993) determined that $\sigma_{f}>1+\frac{\tau_{m}}{\tau_{s}}$ and a ratio τ_*m*_/τ_*s*_ of at least 10 is required. Actually, because of our newly derived learning rule (Equation (11)), we also require σ_*f*_ < 1+σ_*s*_, and best results are now achieved with σ_*f*_ = 1. For greater σ_*f*_, the system is too unstable, and for smaller values, the learning of σ_*s*_ slows down because the neurons are in non-rhythmic behavior.

#### 3.1.1. Inhibition influence on plasticity

**Figure 2:**
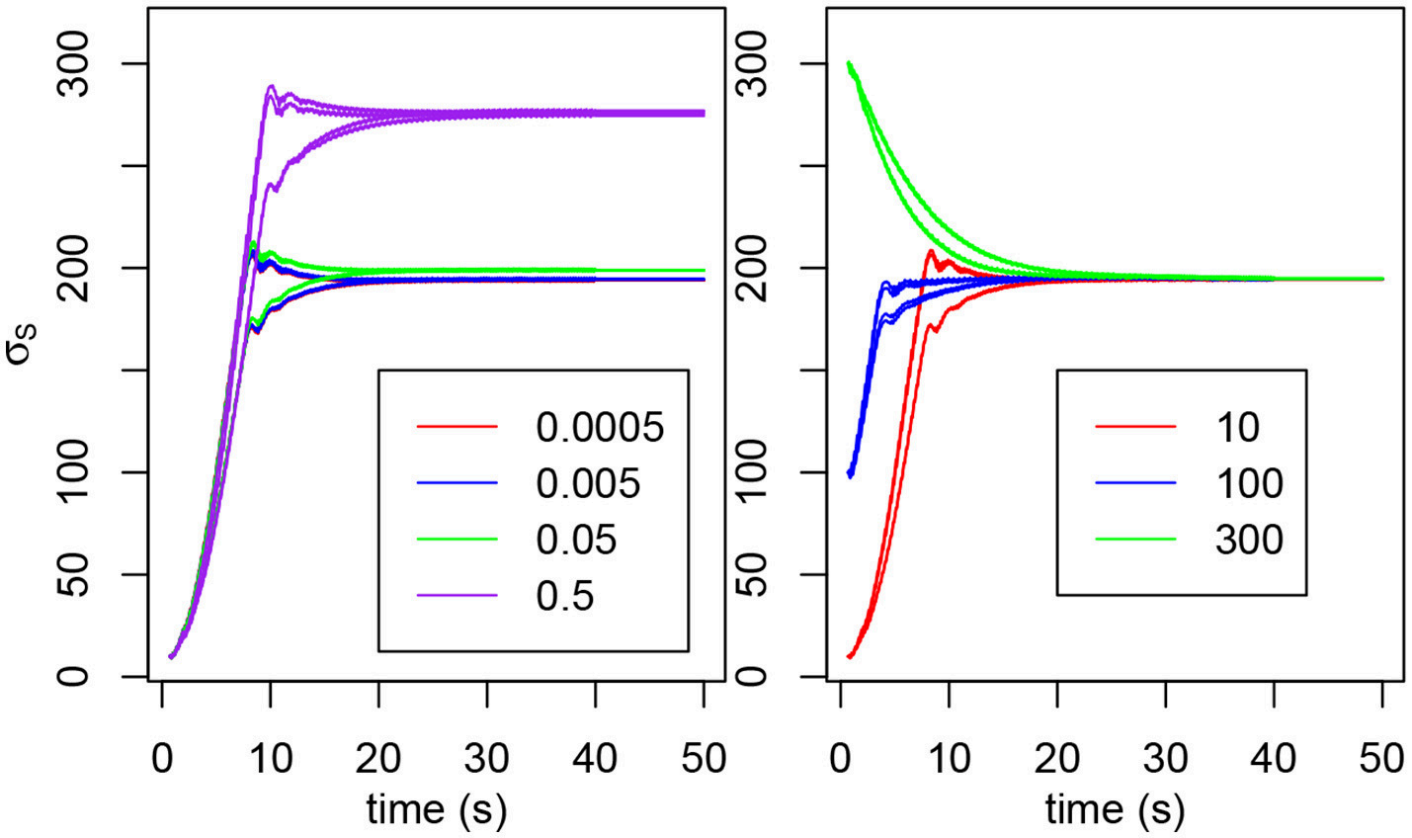
**(Left)** Evolution of σ_*s*_2*E*__, σ_*s*_2*F*__, σ_*s*_3*E*__, and σ_*s*_3*F*__ for various values of *W*. The initial value is 10 for each σ_*s*_. **(Right)** Evolution of σ_*s*_3*E*__ and σ_*s*3*F*_ for various initial values (in red, 10; in blue, 100; in green, 300). The σ_*s*_ have not been distinguished because we're only interested in the tendency and not in the individual behaviors.

We previously stated that the natural frequency of the oscillator is determined by τ_*m*_, τ_*s*_, σ_*f*_ and σ_*s*_ only. However, the natural frequency also depends on *W*. The higher *W*, the lower the frequency, hence the higher σ_*s*_ needs to be to compensate. Figure 2 shows that the value of *W* influences the final value of σ_*s*_ and below *W* = 0.05, the result is roughly the same. We can observe a slight demarcation for *W* = 0.05 and above. For *W*≥1, the system isn't able to oscillate (Rowat and Selverston, 1997).

The initial value of σ_*s*_ doesn't change the final value reached (see Figure 2). For very high or very low values, the final σ_*s*_ may never be reached if the interaction does not last long enough.

#### 3.1.2. Effect of afferent sensory feedbacks on plasticity

**Figure 3:**
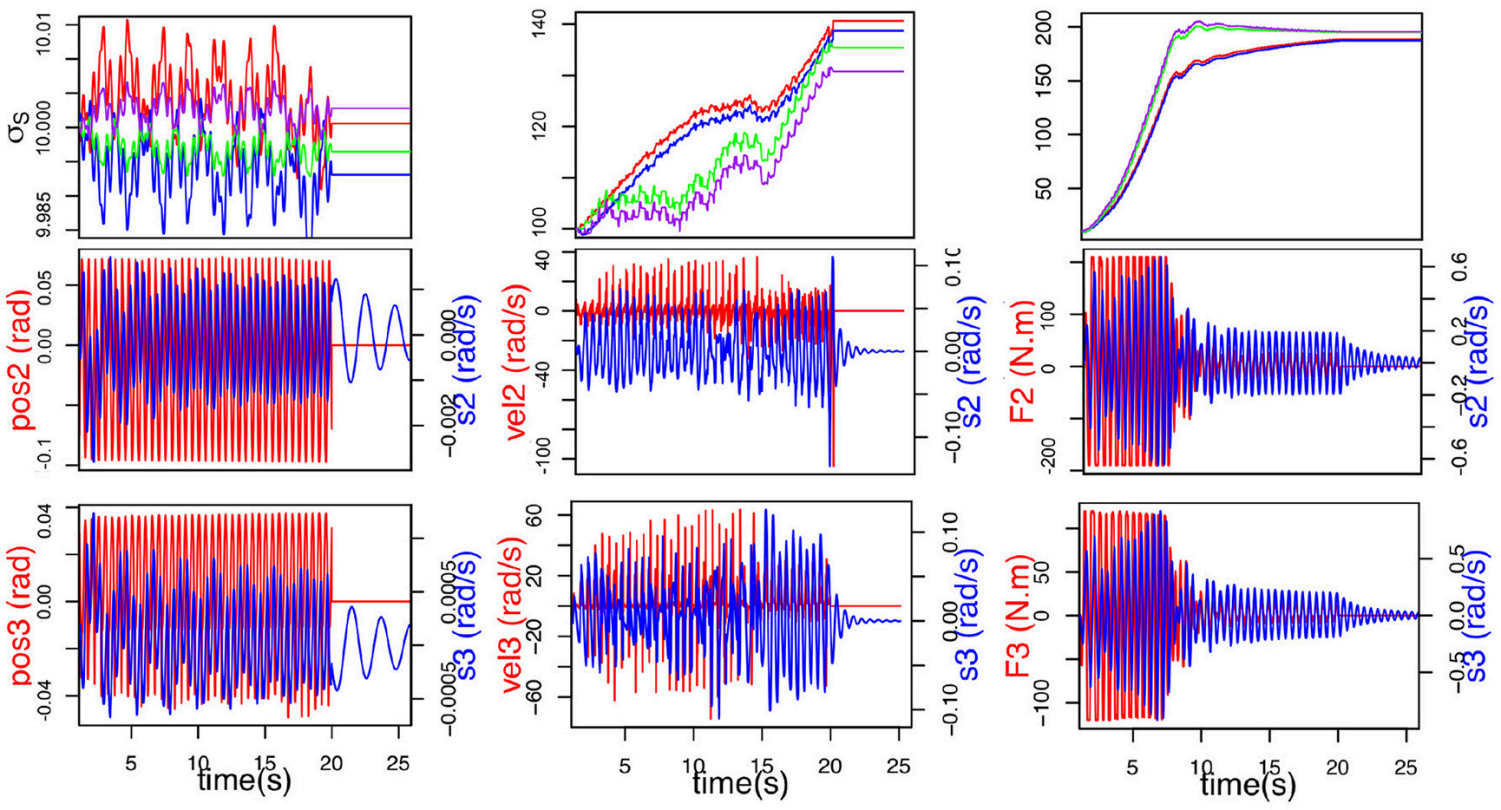
Comparison of different efferent articular signals to the CPG during handshaking: position **(Left)**, velocity **(Middle)**, and torque **(Right)**. Evolution of σ_*s*_ (top) (σ_*S*2*E*_ (blue), σ_*S*2*F*_ (red), σ_*S*3*E*_ (purple) and σ_*S*3*F*_ (green)), *F*(*t*) in red (middle and bottom), and articular velocity in blue.

Tests were carried out to determine which articular sensory information is best suited to our purpose and yields the best result in term of synchronization. Figure 3 shows the comparison between articular position, articular velocity and articular torque as feedback. Position and velocity feedback offer very bad results. Both are neither able to adapt nor synchronize in spite of our best attempts to find better parameter values. Finally force feedback shows the best results. Furthermore, handshaking is a social gesture and as such, provides information about the interaction partner: firmness of grip, strength, vigor. This data can be used to infer personality traits (Chaplin et al., 2000) and can only be sensibly obtained from force feedback. So, the torque measured in the joint will be the afferent input of our CPG for synchronization.

### 3.2. Analyze of the simulated handshake

The simulation lasts 50 s. The interaction starts at *t* = 0.68*s* and lasts until *t* = 40*s* when the gripper opens and releases the ball. In this case, only frequency adaptation (σ_*s*_ learning) is enabled, ϵ and *A*_*f*_ remain constant. The parameters used for the simulation are as follows: ϵ = 0.02 for the shoulder CPG (joint 2), ϵ = 0.03 for the elbow CPG (joint 3), τ_*m*_ = 0.35, τ_*s*_ = 3.5, *W* = 0.005, σ_*f*_ = 1.0 and *A*_*f*_ = 0.05.

#### 3.2.1. Emergence of synchrony in handshaking

**Figure 4:**
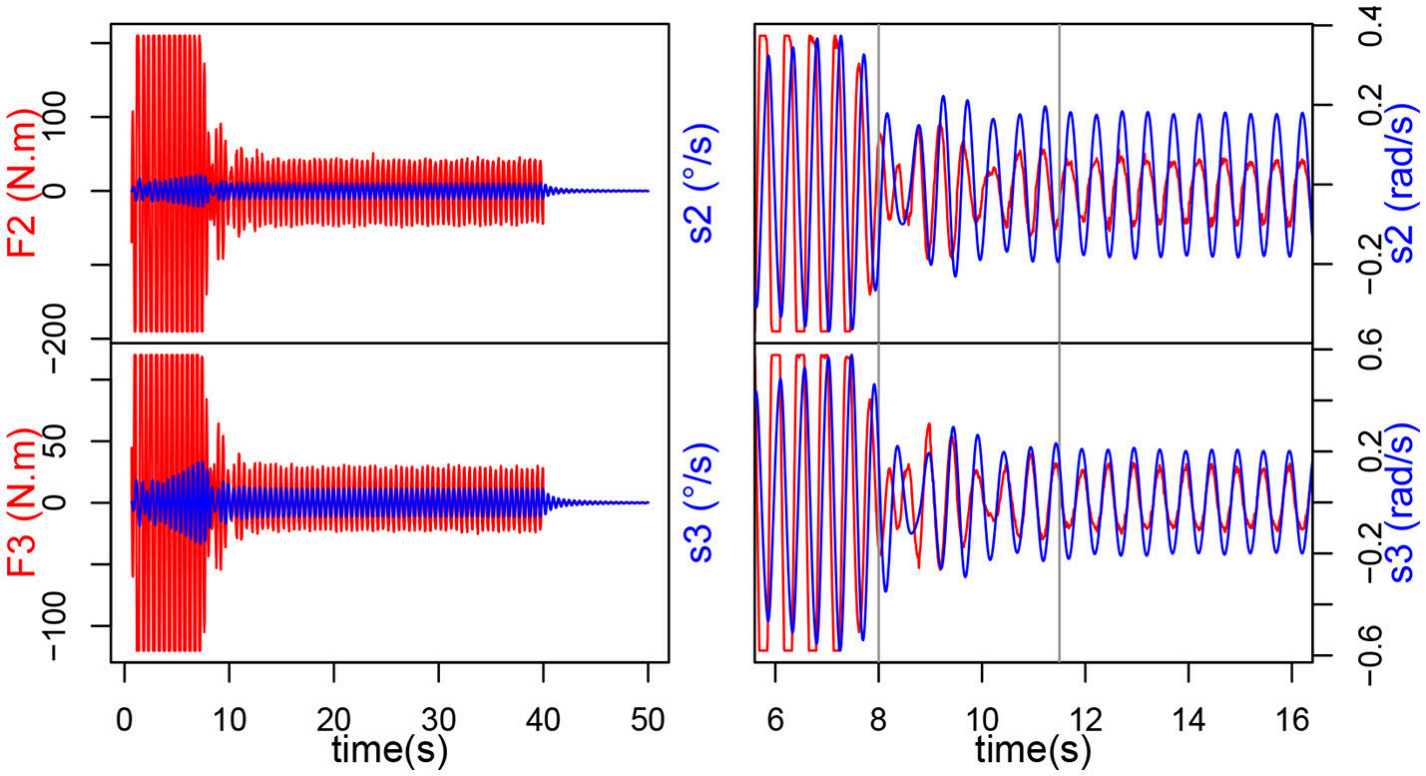
Evolution of torque *F*_*i*_ (input of the CPG *i*) and *s*_*i*_ control speed, for each joint during the human-robot handshake.

**Figure 5:**
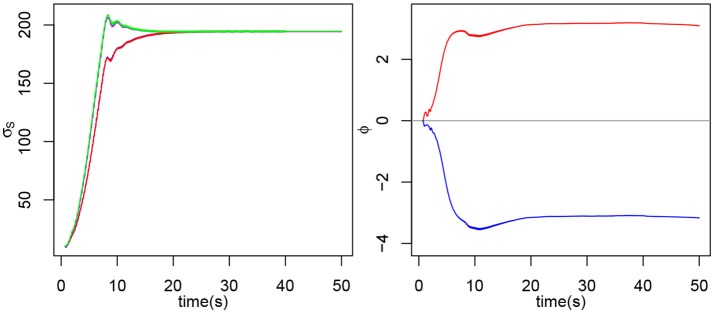
On the **left**, evolution of σ_*S*2*E*_ (blue), σ_*S*2*F*_ (red), σ_*S*3*E*_ (purple) and σ_*S*3*F*_ (green). Note that the two σ_*s*_ for each joint are completely merged, so only one is clearly visible on the plot. On the **right**, evolution of ϕ_3*E*_ and ϕ_3*F*_ during the experiment for the extensor and flexor of the second joint.

The simulated act of handshaking can be divided into four phases among which two specific phases appear showing the emergence of synchronization of movement during contact :

- **Preparation phase**. We won't dwell on this phase, as it offers nothing of interest to this work. At *t* = 0*s*, the arm is at rest. Between *t* = 0 and *t* = 0.68*s*, the arm raises toward the ball which places itself inside the gripper.
- **Transitory phase: contact and learning synchronization**. When the interaction starts, i.e., when the ball starts moving up and down, we can observe, in Figure 4, a massive increase in the torque measured in the joints (200 N.m for joint 2 and 120 N.m for joint 3). The magnitude stays the same for roughly 7 s, while the intrinsic frequency of the oscillator changes until it matches the input frequency. This can be further evidenced by observing the speed command *s* sent to the joints, or even the evolution of σ_*s*_ (see Figure 5). When the interaction starts, they start increasing, all following the same direction, though some are slightly slower than others, they finally catch up around *t* = 20 s. This phase offers two distinctive behaviors: when the force is saturated and the σ_*s*_ increase rapidly; when the force exerted has decreased but *F* and *s* still aren't synchronized and the σ_*s*_ slow down, hinting at stabilization.
- **Locking phase: mutual synchronization**. When the transitory phase is over, the force exerted on the arm decreases and *F* and *s* can be observed to be perfectly synchronized and in phase (Figure 4). From then onwards, the torque amplitude stays mainly stable at 50 N.m for joint 2 and 25 N.M for joint 3, this shows that the arm learned the movement, it oscillates at the right frequency on its own and the ball isn't forcing on it so much. Besides, we can observe that the σ_*s*_ also reach stability, from *t* = 12*s* onwards (by considering a response time at 5% of the final value). The σ_*s*_ for both joints are now completely merged and stable around 192.
- **Withdrawal phase**. Finally the interaction stops at *t* = 40*s* and the ball is released so there isn't any force exerted on the arm. We can see that the arm goes on oscillating at the frequency learned during the interaction, though with a smaller amplitude. The σ_*s*_ also remain stable, showing that the new value has indeed been learned. This oscillation could be stopped by setting the value of σ_*f*_ below 1.

#### 3.2.2. Inter-limb coordination

Inter-limb coordination can be observed thanks to ϕ_*E*_ and ϕ_*F*_ which represent the phase difference of the flexor and extensor motoneuron output, respectively of both CPGs (see Figure 5):

(18) $$\begin{array}{r} \phi_{\left\{E,F\right\}}=\theta \left(V_{2_{\left\{E,F\right\}}},y_{2_{\left\{E,F\right\}}}\right)-\theta \left(V_{3_{\left\{E,F\right\}}},y_{3_{\left\{E,F\right\}}}\right) \end{array}$$

with θ(*V, y*) the phase of the CPG:

(19) $$\begin{array}{r} \theta \left(V,y\right)=\text{sign}\left(V\right)\text{acos}\left(\frac{-y}{\sqrt{V^{2}+y^{2}}}\right) \end{array}$$

Both ϕ start at *t* = 0. Similarly, to our previous observations, during the transitory phase, ϕ_3*E*_ increases while ϕ_3*F*_ decreases. After that, the ϕ reach stability around π and −π, from *t* = 20*s* onwards and retain the same value after the interaction stops at *t* = 40*s*.

#### 3.2.3. Dynamic stability of synchronization

**Figure 6:**
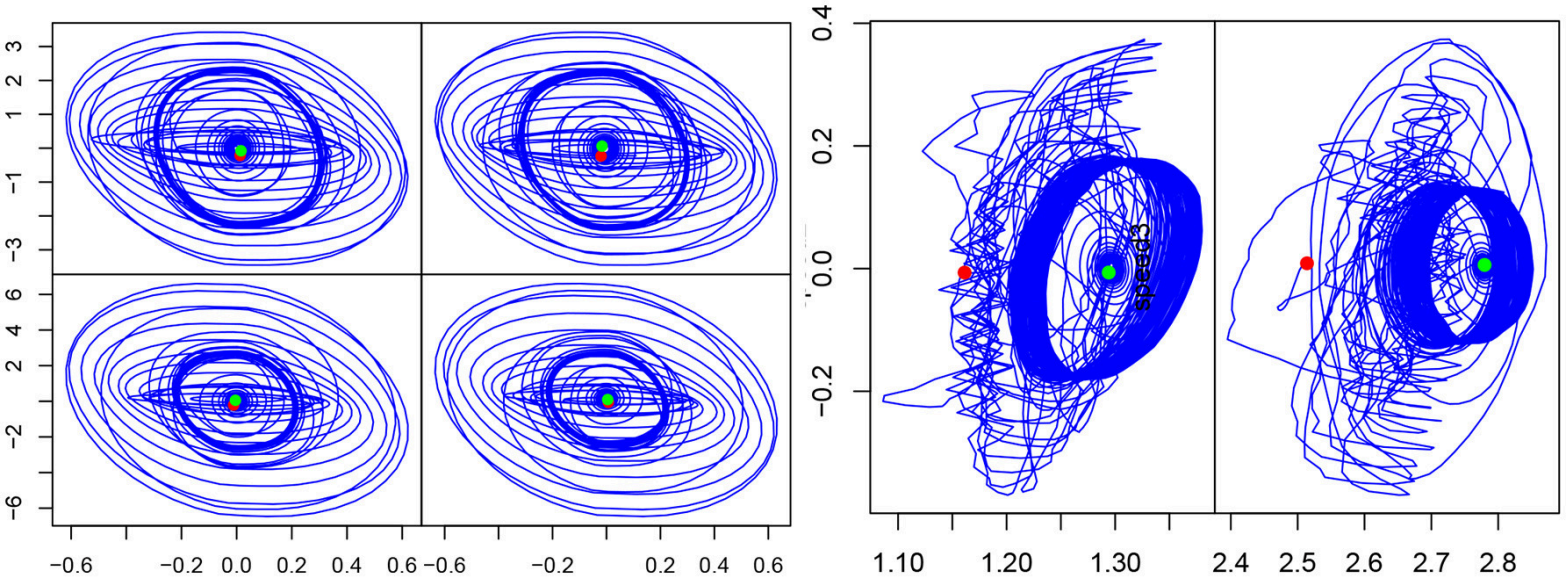
**(Left)** Phase portrait in the (*V, y*) plan for each CPG. **(Right)** Phase portrait (velocity vs position) of the second and third articulations, during the same simulation. The red dot indicates the start and the green one, the end of the experiment.

Dynamic stability of synchronization can be observed through the phase portrait of the CPGs (*V*-*y*) and robot articulations (angular velocity-angular position). On the CPG output phase portraits (see Figure 6), three different cycles can be observed. First, the starting cycle (most inner circle), when the rhythmic cells oscillate at their own intrinsic frequency. Then, the interaction cycle (most outer circle) when the human and the robot are interacting. Finally, the middle circle is the end cycle. On the other hand, on the position-velocity phase portraits we can clearly distinguish two cycles. The outer cycle corresponds to the interaction part, while the inner cycle is the “arm released” part. The cycle does not change in shape, but changes in size (due to the amplitude decreasing) when the arm is released. This cycles apparition shows that the system is stable, and thus the frequency is learned.

### 3.3. Plasticity leads to frequency adaptation

In this study, the frequency of the ball movement varies following Heaviside functions simulating different types of human handshakes: 2 Hz between 0.68 and 35 s, then 1 Hz between 35 and 70 s, then 2 Hz between 70 and 100 s, finally 2.5 Hz between 100 and 120 s. To demonstrate the importance of frequency adaptation for synchronization, a first simulation was run without learning σ_*s*_ (σ_*s*_ would thus remain constant at 10), while a second was run with σ_*s*_ learning enabled.

**Figure 7:**
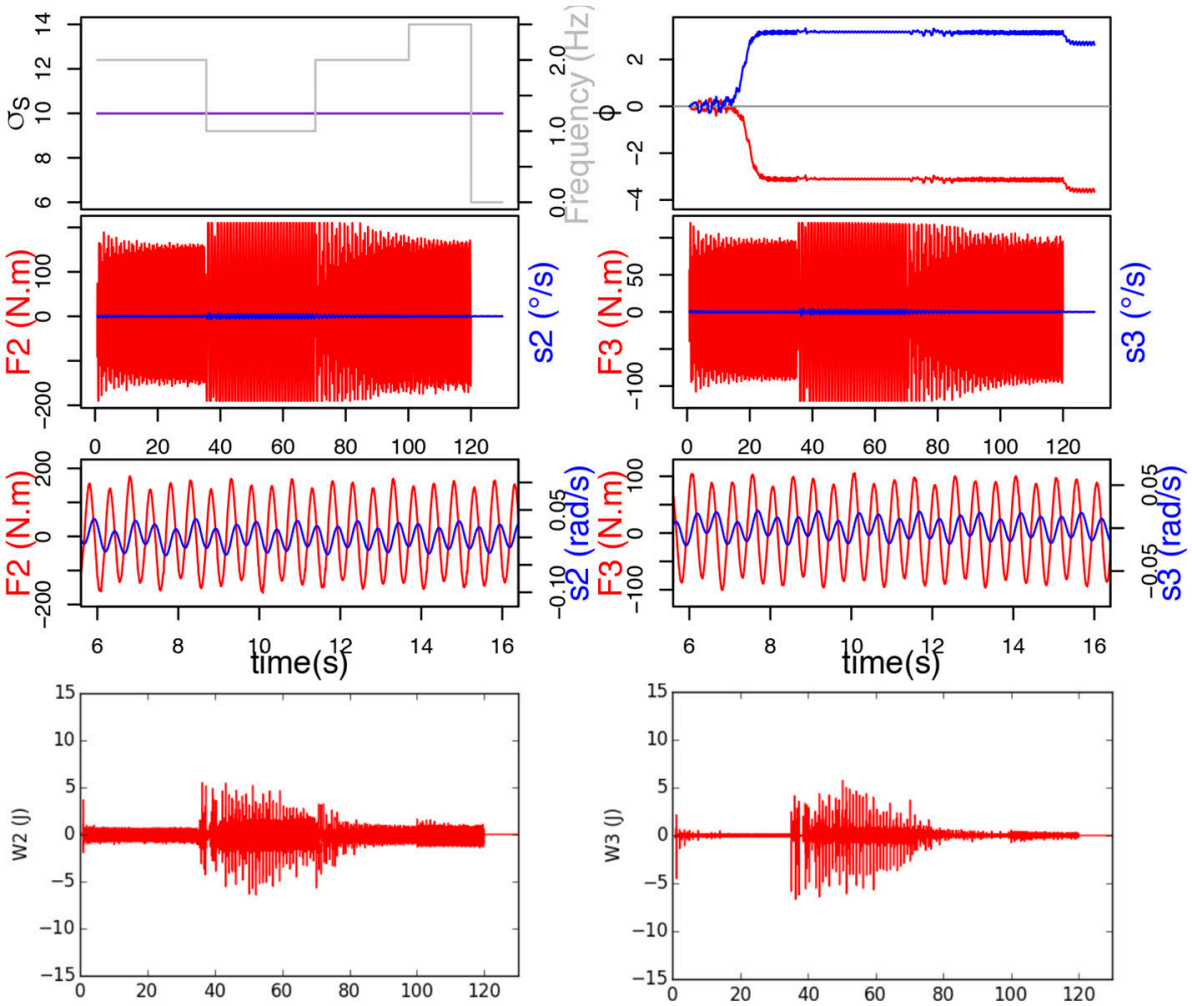
Here, σ_*s*_ doesn't learn and remains constant at 10. Top figure represents the evolution of σ_*s*_, frequency of *F* and ϕ. Below are the force applied on the joint in red and send velocity in blue, and at the bottom, the mechanical work provided by each joint.

Results show that the GPG controller which doesn't learn σ_*s*_ synchronizes with the perturbation signal thanks to its property of natural synchronization, but since it doesn't learn the new frequency, it doesn't reach stability, i.e. the system oscillates at the right frequency, but only because the interaction forces it to. The signals *F* and *s* are neither in phase nor in anti-phase, which would be stable regimen. This leads the system to always provide maximum effort throughout the whole interaction, and the force to be constantly saturated. This can be observed on the bottom of Figure 7 by the important mechanical work provided by the joints.

**Figure 8:**
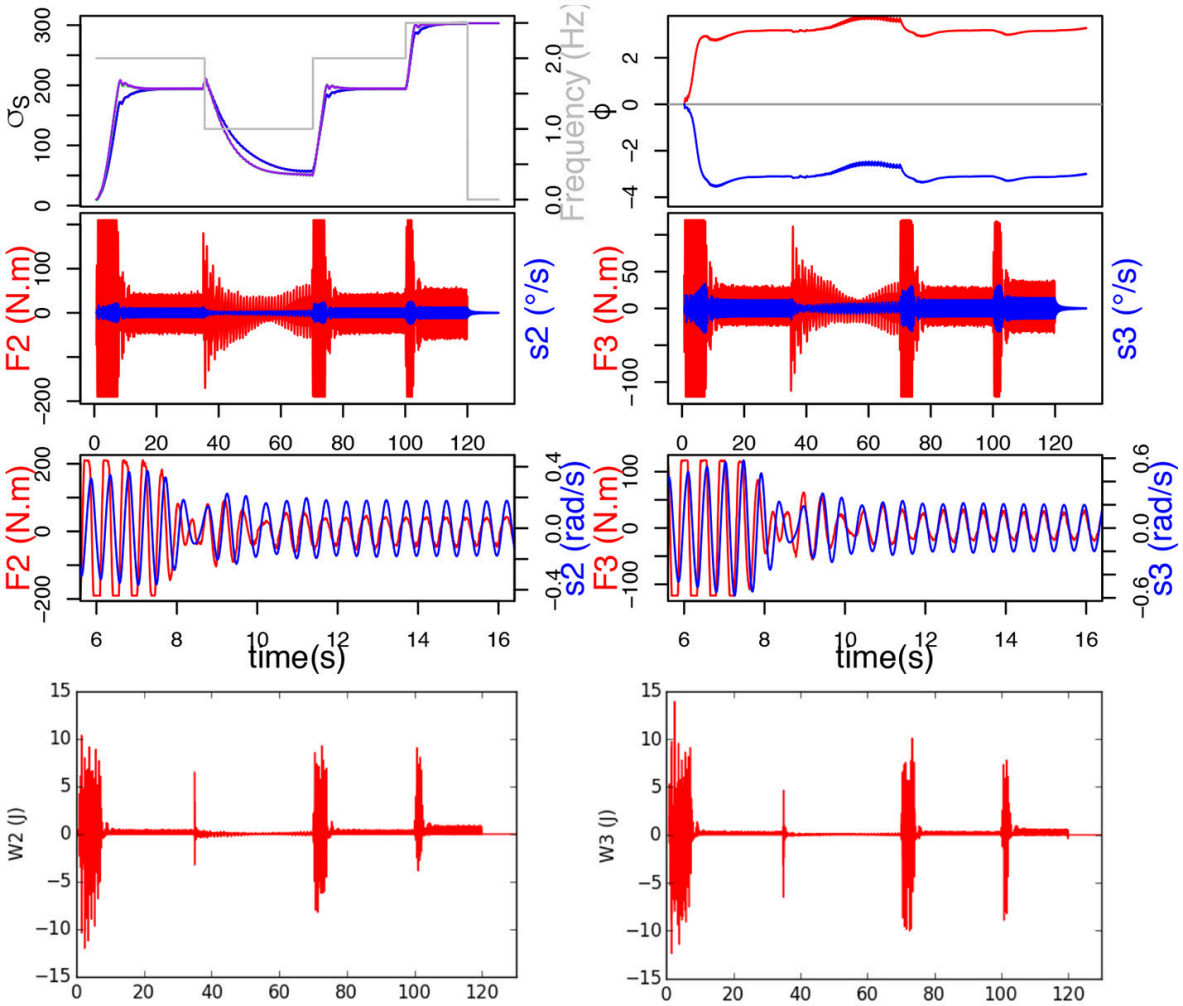
σ_*s*_ learns. Top figure represents the evolution of σ_*s*_, ϕ and frequency of *F*. Below are the torque measured on the joint (red) and send velocity (blue), and at the bottom, the mechanical work provided by each joint.

On the contrary, when the system learns σ_*s*_, we can see that σ_*s*_ indeed adapts to each new frequency and we can observe that the torque and CPG output are synchronized and in phase (Figure 8). The decrease in force, which we previously witnessed in our simple handshaking experiment, occurs here too. In this case, the mechanical work provided by the joints (see bottom of Figure 8) is consequently much less important since the force happens to be saturated only during the transition phases.

### 3.4. Plasticity decreases the energy spend by the robot

It is interesting to study the role of neuronal plasticity (σ_*s*_ and *A*_*f*_) and synaptic plasticity (ϵ), on the energy spend by the robot for synchronization. Since last section has shown the positive effects of learning σ_*s*_ on the mechanical work provided by the motors, this section won't talk about σ_*s*_ learning any more, which will be always enabled. So when employing the terms *without plasticity*, the reader shall understand *without any plasticity (A*_*f*_ *nor* ϵ *learning) but* σ_*s*_ *learning*.

Again, the frequency of the ball movement varies throughout the interaction: 2 Hz between 0.68 and 35 s, then 1 Hz between 35 and 70 s, then 2 Hz between 70 and 100 s, finally 2.5 Hz between 100 and 120 s. The parameter values are the same as in section 3.2.

Moreover, for the first simulation (without plasticity), *A*_*f*_ = 0.05, ϵ = 0.01 for joint 2 and ϵ = 0.02 for joint 3. For the second simulation (with neuronal plasticity), λ = 2.10^−3^, $\mu_{2}=5.10^{-6}$ and $\mu_{3}=8.10^{-6}$.

Results from simulations are evaluated first energetically and second by synchronization time. To calculate the power consumption of the system, we compute the work provided by each joint with following equation:

(20) $$\begin{array}{r} W=\underset{t}{\sum }|F_{t}\Delta \theta_{t}| \end{array}$$

The synchronization time is defined by the 5% response time for both σ_*s*_ to reach the stability value, for each different frequency value.

**Figure 9:**
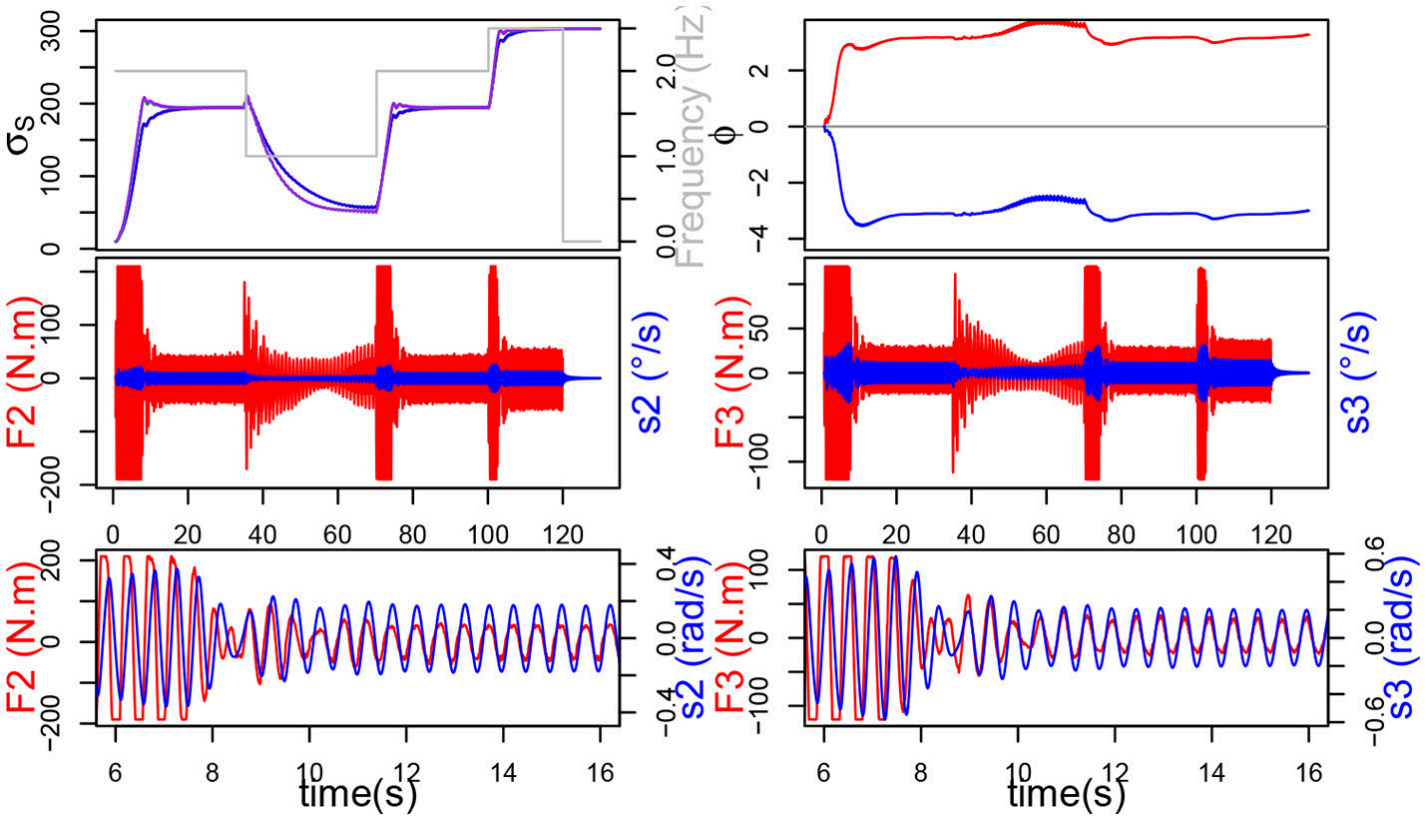
On the left, the simulation without plasticity. On top, the evolution of σ_*s*_ and ϕ. On the bottom, in red the force exerted on each joint and in blue the command velocity.

First, it should be noticed from Figure 9 that the system without plasticity doesn't do too well in the lower frequency 1 Hz. Indeed, after decreasing, the force increases again and the σ_*s*_ of the different joints never merge. These two phenomena, which by the way are also to be found in the simulation with only *A*_*f*_ plasticity, are due to the value of ϵ which, while suitable for the other frequencies, is too small to get good results at 1 Hz.

**Figure 10:**
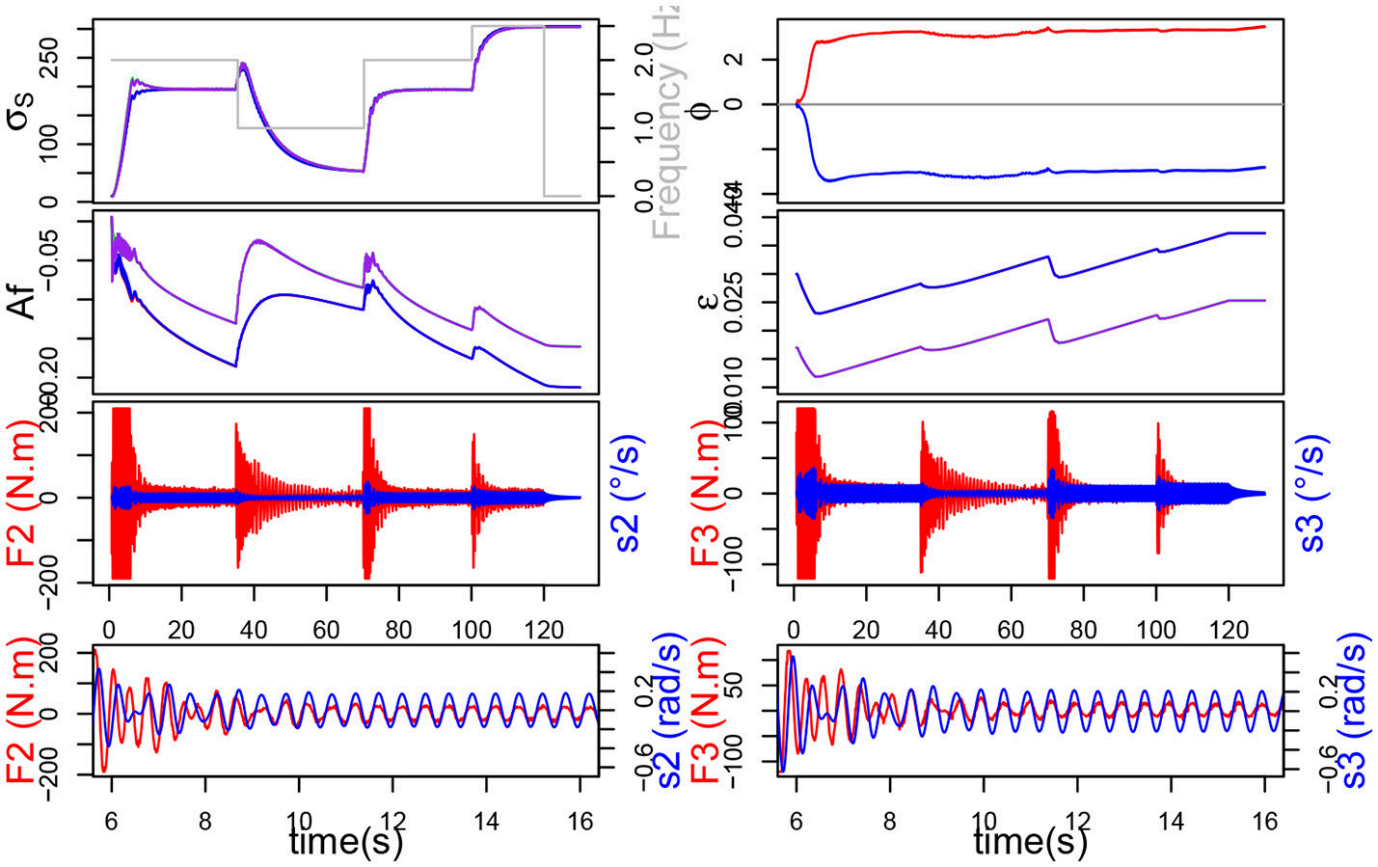
The simulation with neuronal plasticity. On top, the evolution of σ_*s*_ and ϕ, below the evolution of *A*_*f*_ and ϵ. On the bottom, in red the force exerted on each joint and in blue the command velocity.

We can also see that the force applied on the joints of the system is much lower when plasticity is applied (Figure 10). The force also decreases faster, this can be correlated with the evolution of the σ_*s*_ which is steeper during the transitions but slows down a little before reaching the new stability value. Those observations suggest that, although the synchronization times may appear similar in the Table 1, the transitory phase might be shorter, and hence synchronization faster, with plasticity. Furthermore, let us remark that, although the synchronization time appears much smaller without ϵ plasticity for 1 Hz, the validity of the measure for the other two cases could be discussed, since the σ_*s*_ never merge.

**Table 1:** Comparison table for various simulations with or without neuronal plasticity.

**Frequency →**	**2 Hz**	**1 Hz**	**2 Hz**	**2.5 Hz**	
**Plasticity**	τ_1_ **(s)**	τ_2_ **(s)**	τ_3_ **(s)**	τ_4_ **(s)**	*W*_2_ **(J)**	*W*_3_ **(J)**
None	12.82	25	6.3	6.5	4,144.69	3,683.31
*A*_*f*_	12.82	25	6.3	7	3,459.06	3,553.71
ϵ	6.82	30	5.7	8.5	2,744.99	2,041.26
*A*_*f*_+ϵ	6.82	32	5.5	8	2,101.04	1,737.47

*τ_i_ is the time required so that σ_s_ stabilizes at the i^th^ frequency change*.

In Table 1, *W*_*i*_ represents the sum of the mechanical work provided by joint *i* during the simulation (synchronization times have been already explained so we won't dwell on the subject any further). We can see that learning the amplitude *A*_*f*_ decreases the work noticeably for joint 2, but only slightly for joint 3. Besides, learning ϵ alone decreases the work further and the artifacts mentioned before disappear. Finally the association of both ϵ and *A*_*f*_ learning yields the best results by virtually halving the original work value.

## 4. Discussion and conclusion

In this paper, we implemented Hebbian mechanisms in a bio-inspired CPG, thus enabling it to learn to synchronize with an external signal. Furthermore, we used this plastic CPG to control a simulated robotic arm which had to learn to synchronize its oscillatory movements to the frequency of an external force applied on its effector.

We also underlined the relevance of force feedback, which not only yields much better results than velocity and position feedback but is also able to provide useful information, such as firmness of grip, strength, vigor. Such data, as evidenced in Chaplin et al. (2000) can be used to assess personality traits of the handshaking partner. This knowledge would allow the robot to adapt to different personalities (introvert, extrovert…), and thus make the interaction more enjoyable.

The analysis of synchronization phenomena clearly shows two main phases: the transitory phase where the system adapts and learns and the permanent phase where the system has retained the learning and is stable. Our best synchronization time is 6.82 s which is quite long for a handshake. Let us underline that we did not put the system in the best conditions to achieve faster coordination, the initial σ_*s*_ (10, 0.44 Hz) being quite different from the final value (192, 2 Hz). Our main concern here was to show the capacity of the CPG to adapt even to very different frequencies from its own., the Mico robot is not compliant and thus offers too much resistance to any perturbation. As a matter of fact, most robots are not designed for such tasks: lacking force/torque sensors, and the robot controllers can also be inadequate, providing only position control. So, putting the CPG in better initial conditions and using a more compliant robot would undoubtedly lead to a much faster synchronization.

Moreover, we demonstrated the importance of neuronal and synaptic plasticity which leads to a natural, global synchronization and adapts the neuronal architecture to a wider range of arm dynamics in physical interaction. On the one hand, we showed that learning σ_*s*_ is paramount to have an adaptive system robust to frequency changes. On the other hand, this system can be improved further by learning the amplitude *A*_*f*_ and the synaptic weight ϵ and hence considerably decreases the power consumption. We showed that local plasticity mechanisms trigger the emergence of a global adaptive stable behavior. In conclusion, it is our belief that plasticity is essential in designing a versatile and reliable bio-inspired controller.

Concerning the methodology followed in this work, it could obviously be argued that a single neuron can simply be used for each joint instead of a whole CPG. Let us answer that we wish to be as biologically close as possible, so our approach uses a mesoscopic model based on Rybak and McCrea's work (Rybak et al., 2006). Apart from that, a CPG offers more possibilities than a simple neuron due to its structure that creates a more robust and stable attractor.

Furthermore, the CPG model used for the rhythmic arm movement during physical interaction is the same as for walking, proving its versatility. On top of that, it should be noted that no dynamic model of the robot was used to control it. The dynamic control of the rhythmic movements relies solely on the natural synchronization abilities of the CPG. This makes the CPG-based control particularly interesting since it can very easily be adapted to another set of joints. Indeed, our simulation was only concerned with handshaking but this plastic CPG model could be applied to any rhythmic movements: walking, waving, cleaning, drumming.

Here, we use the well-known slave-master paradigm where one actor of the interaction imposes its frequency upon the other but we're also interested in studying how two robots would adapt to each other. In the future, we plan on extending the CPG architecture to more than two joints. Using a simulator obviously entails its share of limitations and our oversimplified handshake oversees a lot of subtleties present in human-robot interactions. Our controller will be validated with a real compliant robotic arm interacting with a human. Additionally, in order to better understand handshaking and hence, better reproduce it with robots, we will continue our study of handshaking, its synchronization phenomena and societal impact by performing human psychological/physiological studies.

Our code can be found at http://doi.org/10.5281/zenodo.1222100

## Author contributions

LC, PH, and MJ wrote the paper. LC introduced frequency learning. MJ ran the simulations and introduced amplitude and synaptic weight learning. PH supervised the work.

### Conflict of interest statement

The authors declare that the research was conducted in the absence of any commercial or financial relationships that could be construed as a potential conflict of interest.

## Appendix

### Mathematical details for section 2.2.2

Let us recall the Rowat Selverston equations:

(21a) $$\begin{array}{r} \tau_{m}\dot{V}+V-A_{f}\tanh\left(\frac{\sigma_{f}}{A_{f}}V\right)+q=0 \end{array}$$

(21b) $$\begin{array}{r} \tau_{s}\dot{q}=-q+\sigma_{s}V \end{array}$$

Equation (21a) can be differentiated, yielding:

(22) $$\begin{array}{r} \tau_{m}\ddot{V}+\dot{V}-\sigma_{f}\left(1-\tanh^{2}\left(\frac{\sigma_{f}}{A_{f}}V\right)\right)\dot{V}+\dot{q}=0 \end{array}$$

and $\dot{q}$ replaced by the expression given in equation (21b):

(23) $$\begin{array}{r} \tau_{m}\ddot{V}+\dot{V}-\sigma_{f}\left(1-\tanh^{2}\left(\frac{\sigma_{f}}{A_{f}}V\right)\right)\dot{V}+\frac{1}{\tau_{s}}\left(\sigma_{s}V-q\right)=0 \end{array}$$

Then we replace *q* by its expression from equation (21a):

$$\begin{array}{rcl} \tau_{m}\ddot{V} & + & \dot{V}-\sigma_{f}\left(1-\tanh^{2}\left(\frac{\sigma_{f}}{A_{f}}V\right)\right)\dot{V}+\frac{\sigma_{s}}{\tau_{s}}V+\frac{1}{\tau_{s}}V+\frac{\tau_{m}}{\tau_{s}} \end{array}$$

(24) $$\begin{array}{r} \dot{V}-\frac{A_{f}}{\tau_{s}}\tanh\left(\frac{\sigma_{f}}{A_{f}}V\right)=0 \end{array}$$

and group the terms:

$$\begin{array}{rcl} \tau_{m}\ddot{V} & + & \left(\frac{\tau_{m}}{\tau_{s}}+1-\sigma_{f}+\sigma_{f}\tanh^{2}\left(\frac{\sigma_{f}}{A_{f}}V\right)\right) \end{array}$$

(25) $$\begin{array}{rcl} \dot{V} & + & \frac{1+\sigma_{s}}{\tau_{s}}V-\frac{A_{f}}{\tau_{s}}\tanh\left(\frac{\sigma_{f}}{A_{f}}V\right)=0 \end{array}$$
